# Multi-omics analysis of thermal stress response in a zooxanthellate cnidarian reveals the importance of associating with thermotolerant symbionts

**DOI:** 10.1098/rspb.2017.2654

**Published:** 2018-04-18

**Authors:** Maha J. Cziesielski, Yi Jin Liew, Guoxin Cui, Sebastian Schmidt-Roach, Sara Campana, Claudius Marondedze, Manuel Aranda

**Affiliations:** King Abdullah University of Science and Technology (KAUST), Red Sea Research Center (RSRC), Biological and Environmental Sciences & Engineering Division (BESE), Thuwal, Saudi Arabia

**Keywords:** transcriptomics, proteomics, heat stress, thermotolerance, *Aiptasia*, oxidative stress

## Abstract

Corals and their endosymbiotic dinoflagellates of the genus *Symbiodinium* have a fragile relationship that breaks down under heat stress, an event known as bleaching. However, many coral species have adapted to high temperature environments such as the Red Sea (RS). To investigate mechanisms underlying temperature adaptation in zooxanthellate cnidarians we compared transcriptome- and proteome-wide heat stress response (24 h at 32°C) of three strains of the model organism *Aiptasia pallida* from regions with differing temperature profiles; North Carolina (CC7), Hawaii (H2) and the RS. Correlations between transcript and protein levels were generally low but inter-strain comparisons highlighted a common core cnidarian response to heat stress, including protein folding and oxidative stress pathways. RS anemones showed the strongest increase in antioxidant gene expression and exhibited significantly lower reactive oxygen species (ROS) levels *in hospite*. However, comparisons of antioxidant gene and protein expression between strains did not show strong differences, indicating similar antioxidant capacity across the strains. Subsequent analysis of ROS production in isolated symbionts confirmed that the observed differences of ROS levels *in hospite* were symbiont-driven. Our findings indicate that RS anemones do not show increased antioxidant capacity but may have adapted to higher temperatures through association with more thermally tolerant symbionts.

## 1. Introduction

Coral reefs are complex and diverse ecosystems that provide habitats for thousands of marine species [1]. Climate change as a result of anthropogenic impacts has long been defined as one of the most serious and potentially fatal stressor to corals, the foundation species of coral reef ecosystems [2]. Rising sea surface temperatures have led to an increase in thermally induced stress, leading to the breakdown of the vital endosymbiotic relationship between corals and the dinoflagellate *Symbiodinium* [3]. The loss of these symbionts, known as bleaching, can have adverse effects on the health of corals and eventually lead to their death.

Corals are globally distributed across habitats with extremely different conditions. In particular, corals in seasonal hot waters, such as the Arabian Gulf or the Red Sea (RS), and in highly thermal variable environments, have demonstrated a capacity to adapt to a large range of temperatures [4]. Corals living in extreme environments also show improved thermal tolerance when compared to counterparts from more thermally stable habitats [5]. The overall resilience of the coral holobiont is dependent on the physiological capabilities of both partners—the coral host and associated *Symbiodinium* [6,7].

Coral–symbiont associations have shown geographical specificity, indicating that environmental selection of symbiosis may exist across regions and hence determine *Symbiodinium* community structure in corals [8]. The importance of *Symbiodinium* in the holobiont's capacity to respond to stressors is evident through corals ability to shuffle symbiont communities during and after stress exposure [9,10]. In particular, *Symbiodinium* in the RS and the Persian/Arabian Gulf (PAG) have shown higher temperature tolerance, and are suggested to be crucial in the overall thermotolerance of the holobiont [11]. However, the extent to which these two partners contribute to thermal tolerance of the holobiont is still poorly understood.

Understanding the underlying mechanisms and the contribution of the two partners that allow enhanced stress tolerance is imperative for future coral conservation efforts, particularly for attempts of assisted evolution [12]. Rapid progress in transcriptomics has indicated regulation of gene expression as a potential mechanism for acclimatization and source of genotypic variation [13]. Studies focusing on various coral species have corroborated key pathways involved in thermal tolerance including protein folding, apoptosis and oxidative stress response [4,14].

Production of reactive oxygen species (ROS) is increased in both cnidarian and symbiotic cells in response to elevated temperatures. The consequent elevation of oxidative stress, primarily owing to the symbiont, is thought to be one of the main causes for coral bleaching [15]. The increased ROS production of the symbiont leaks to the host, leading to cellular damage and initiation of apoptosis in the coral cells [16]. Hence, thermotolerant symbionts have been accredited with lower levels of ROS production during heat stress exposure, therefore reducing direct stress to the coral [17,18]. In turn, a thermotolerant host has a higher threshold for ROS levels through antioxidant responses, allowing sufficient detoxification to prevent bleaching [18].

While a significant amount of information regarding thermotolerance mechanisms has been obtained on a transcriptomic level, investigations into the proteome have so far been sparse. A narrow assortment of papers uses proteomic analysis to elaborate on fundamental cnidarian biology under stress response [19,20]. Easy availability of mRNA data has led to an increased dependence on the transcriptome to answer questions regarding biological response and functionality. However, previous investigations into mRNA–protein dynamics have shown a lack in their expected correlation [21,22]. These low correlations between transcript and protein levels raise the question of whether gene expression is an accurate proxy for the phenotype.

**Figure 1.**
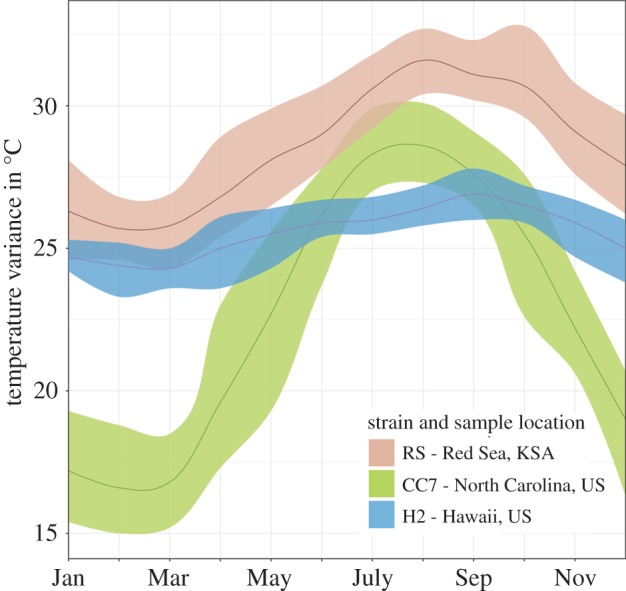
Annual average temperature profiles of sampling locations of the three investigated *Aiptasia* strains originating from North Carolina (Wilmington), Hawaii (Kāne'ohe Bay) and the Red Sea (Al Lith). The average monthly temperature is denoted by a solid line. Shading around lines represent maximum and minimum temperature (temperature data taken for closest location to sampling area from www.seatemperature.org).

In this study, we investigated thermal stress response variations in the transcriptome and proteome of the cnidarian model organism *Aiptasia pallida* (*sensu Exaiptasia pallida* [23]) from three geographically distinct locations with different seasonal temperature profiles (figure 1). We used anemones originating from North Carolina (CC7), Hawaii (H2) and the RS to analyse differences and similarities in heat stress response. Besides their potential genotypic adaptations, the three strains associate with different *Symbiodinium* clades (electronic supplementary material, table S1). We investigated anemone strain specificity in thermal response to determine pathways that appear crucial for the survival in thermal extremes as well as potentially drive resilience. By employing a combined -omics approach, we provide, to our knowledge, the first comparison of transcriptome- and proteome-wide response of a zooxanthellate cnidarian to heat stress.

Our study shows the complexity of the interacting relationship between the cnidarian host and its symbiotic partners. Furthermore, results presented indicate that the thermotolerance of the symbiont plays a crucial role in cnidarian temperature stress adaptations, highlighting the importance of understanding host–symbiont dynamics when attempting to enhance coral bleaching resilience.

## 2. Material and methods

### (a) Growth and maintenance of *Aiptasia* strains

Three *Aiptasia* strains were used in this study, all of them clonal. CC7 originated from North Carolina, NC [24], while H2 was collected from Coconut Island, HI [25]. RS was obtained from Al Lith, Saudi Arabia (20°04′58″ N, 40°07′59″ E).

All *Aiptasia* strains were housed in replicate (two tanks per strain) polycarbonate tanks in a strain-specific manner, and incubated at 25°C and a light intensity of approximately 80 µmol photons m^−2^ s^−1^ on a 12 h : 12 h light:dark schedule. The *Aiptasia* were fed with freshly hatched *Artemia salina* (brine shrimp) nauplii thrice per week.

### (b) Subjecting *Aiptasia* to heat stress

Prior to the heat stress experiments, for each of the studied strains, 12 individuals were chosen and randomly subdivided into smaller (but identical) polycarbonate tanks, which guards against accidental death of stressed individuals during the experiment. These tanks were incubated without food for three days to allow the *Aiptasia* to settle and acclimatize to their new tanks.

For the heat stress experiment, all conditions were identical, save the temperature of the incubators. Experimental tanks were slowly ramped up in temperature from 25–32°C at the rate of 2°C per hour starting from 08.00 and reaching the target temperature by noon to mimic natural conditions. Control tanks were maintained at 25°C. The stress duration lasted from noon until noon of the next day. Samples were collected after 24 h of heat stress at noon, the middle of their daily light cycle.

### (c) Simultaneous extraction of protein and RNA from *Aiptasia* individuals

Four individual biological replicates were picked from control and treatment tanks. Individuals were placed in separate Eppendorf tubes, dried by removing excess water and weighed. Based on its dry weight, a proportional amount of Buffer RLT was added to the Eppendorf tube, and the *Aiptasia* was crushed using a motorized pestle with Kontes RNase-free tips (Kimble Chase, Vineland, NJ) on ice.

RNA was extracted using the RNeasy Mini Kit (Qiagen, Hilden, Germany) with one modification: after crushing anemones in Buffer RLT, the first flow-through (which contains the protein fraction) in the RNeasy spin columns were retained in separate Eppendorf tubes. RNA concentration of the samples was quantified using Qubit 2.0 (Invitrogen, Carlsbad, CA), and quality-checked using Bioanalyzer 2100 (Agilent, Santa Clara, CA).

The protein fraction in the flow-through was cleaned up following a manufacturer-provided protocol to precipitate proteins from Buffer RLT with acetone (Qiagen, Hilden, Germany). Protein concentrations were quantified via Bradford assay using BioPhotometer Plus (Eppendorf, Hamburg, Germany).

### (d) Library construction from total RNA

Total RNA from all samples (*n* = 8 from each strain, i.e. four from control, four from treatment) were initially subjected to a poly-dT selection step, and then used to generate libraries using the Illumina TruSeq RNA Sample Prep Kit (Illumina, San Diego, CA) per manufacturer's instructions. Sequencing was carried out on the HiSeq 2000 platform (Illumina, San Diego, CA), producing a grand total of 874 million reads.

### (e) Identification of differentially expressed transcripts

The reads were mapped to the gene models from *Aiptasia* [26] with the use of Kallisto v0.42.4 [27]. To reduce ambiguity in read-mapping downstream, a custom Python script was written to pick the longest isoform on a per-loci basis, leading to the decrease of overall *Aiptasia* gene models from 29 269 to 27 504.

Based on the transcripts per million (TPM) values produced from Kallisto, sleuth [28] was used to identify genes that were differentially expressed (Benjamini–Hochberg-corrected *p* value < 0.05) under heat stress relative to control conditions.

### (f) Sequencing the proteome of *Aiptasia*

Protein fractions were labelled using an iTRAQ labelling method according to manufacturer's protocol. Purified, digested and labelled samples were taken through high performance liquid chromatography (HPLC) for elution of peptides into 15 fractions and then pooled down to five fractions. All samples were then run through liquid chromatography-tandem mass spectrometry (LC-MS/MS) to produce spectral quantification of peptides.

The NanoLC-MS/MS analysis was performed using an online system consisting of a nano-pump UltiMate 3000 UHPLC binary HPLC system (Dionex, Sunnyvale, CA) coupled to a Q-Exactive HF mass spectrometer (Thermo Fisher Scientific, Darmstadt, Germany). Peptides were resuspended in 20 µl of sample buffer (3% ACN, 0.1% formic acid) and 2 µl was injected into a pre-column 300 µm × 5 mm (Acclaim PepMap, 5 µm particle size). After loading, peptides were eluted to an Acclaim PepMap100 C18 capillary column (75 µm × 15 cm, 100 Å, 3 µm particle sizes). Peptides were eluted into the MS at a flow rate of 300 nl min^−1^, using a 40 min gradient from 5% to 40% mobile phase B. Mobile phase A was 0.1% formic acid in H_2_O and mobile phase B was 80% acetonitrile and 0.1% formic acid. The MS was operated in positive and data-dependent mode, with a single MS scan (350–1400 *m*/*z* at 60 000 resolution (at 200 *m*/*z* in a profile mode) followed by MS/MS scans on the 10 most intense ions at 15 000 resolution. Ions selected for MS/MS scan were fragmented using higher energy collision dissociation (HCD) at normalized collision energy of 28% and using an isolation window of 1.8 *m*/*z*.

MaxQuant software 1.5.2.8 [29] was used for peptide and protein identification. Raw files were processed using the MaxQuant software interlinked with the local MASCOT server (Matrix Science, London, UK). Mascot searches were done against *Aiptasia* gene models; contaminants were excluded. Precursor mass tolerance of 20 ppm and a fragment ion mass tolerance of ±0.5 Da were used. In addition, carbamidomethyl modification on cysteine residues was used as a fixed modification, oxidation on methionine residues as variable modifications and the decoy database was selected. Further stringency was applied for a robust identification by using minimum peptide length of seven and setting the false discovery rate of 0.01.

### (g) Identification of differentially expressed proteins

Data processing followed a method outlined previously [30] that allows for within- and across-run comparisons without the use of a reference sample. Briefly, samples that failed to produce good quality spectral readings were removed (CC7 25 no. 1, H2 25 no. 2, RS 32 no. 2). Spectral values were log_2_-transformed, and subsequently a correction factor that controls for loading effect (which affects the absolute amounts of peptides detected by the machine) was subtracted from each run. Another correction factor was subtracted across runs on a per-gene basis (termed ‘median-polished log_2_’ in Herbrich, Cole [30]), allowing comparisons of relative expression of the same protein across samples.

Relative expressions and standard errors were then calculated for each protein in every strain-temperature combination. To remove rarely detected and low-quality peptide data, filters were instituted on a per-strain-temperature basis: only peptides detected in ≥2 iTRAQ technical replicates and ≥1 biological replicates were retained. For each strain, pairwise log_2_-fold changes in protein levels were calculated as the difference between mean log_2_-expression at 32°C and mean log_2_-expression at 25°C. We initially set a permissive fold change threshold of 1.2× in either direction to identify proteins that were differentially expressed under heat stress, paralleling published proteomics efforts on marine organisms [31].

To provide further statistical insight into our results, we subjected the protein values to two more stringent checks. Firstly, *t*-tests comparing the relative expression values of the same protein across temperatures were carried out on proteins that fulfilled the filters mentioned previously (≥2 iTRAQ technical replicates and ≥1 biological replicates, *n* = 4296 proteins). Secondly, inspired by a recent study on the same organism [32], we implemented a multivariate generalized linear model (GLM) to model whether protein expressions were affected by strain or temperature, or a combination of both factors, via a Python script. All calculated *p* values were subsequently corrected for multiple testing [33].

### (h) Correlating transcript and protein expression patterns

Based on the processed transcript and protein data, two correlations were investigated: whether absolute transcript values and absolute protein values were tightly linked, and whether relative changes of transcript expression were similar to that of protein expression under thermal stress.

A complication to both analyses was that some detected peptides (approx. 10% of all) could originate from multiple gene models. To reduce ambiguity, these peptides were removed from the dataset, and subsequently plotted with Python.

### (i) Functional enrichment of differentially expressed gene models

Since some detected peptides could originate from multiple gene models, if all gene models that could give rise to the differentially expressed peptide were included in the functional analysis, it would result in the over-representation of functional categories associated with the peptide. To remove this bias, a representative gene model was chosen per ambiguous peptide via a Python script.

This complication was not present for the transcript data. Functional enrichment was carried out with topgo [34] with default settings; however, the background gene set (universal set) was set as gene models with detectable expressions. This precaution, which was more applicable to the protein dataset than the transcript dataset, prevents the analysis from being biased towards gene ontology (GO) terms that are associated with expressed gene models. GO terms with *p* < 0.05 and occurring ≥5 times in the background set were considered significant. Multiple testing corrections were not carried out, as the tests were considered to be non-independent [34].

### (j) Biochemical assaying of reactive oxygen species

ROS production of symbionts *in hospite* during heat stress exposure was measured by repeating the stress experiment as described previously. After 24 h of heat stress, anemones were placed in individual Eppendorf tubes and incubated in 500 µl of seawater containing 5 µM CellROX Green Reagent (Thermo Fisher Scientific, Waltham, MA) for 2 h in the dark at both temperatures. Anemones were washed twice in phosphate buffered saline and crushed in 400 µl of cell lysis buffer (200 mM TRIS-HCl pH 7.5, 2M NaCl, 0.1% Triton 20%). Homogenates were spun down at 14 000*g* for 3 min, and 150 µl of the supernatants were loaded into a 96-well black clear-bottom plate. Fluorescence intensity of CellROX dye was measured at excitation of 468 nm and emission at 520 nm using SpectraMax Paradigm Multi-Mode Detection Platform (Molecular Devices, CA).

For the measurements of ROS production in *Symbiodinium* isolates, symbionts were extracted from four anemones of each strain prior to heat stress. Anemones were crushed in 1 ml autoclaved seawater containing kanamycin, ampicillin and streptomycin (50, 100 and 50 µg ml^−1^ respectively) using a Wheaton tissue grinder. The homogenates were filtered through a 40 µl mesh to remove larger cellular debris. The resulting filtrates were equally divided into two 500 µl fractions, then incubated separately at 25°C (control) and at 32°C for 24 h.

Post-incubation, symbiont counts were performed on 100 µl of the filtrates using a Guava easyCyte 8HT benchtop flow cytometer (Millipore, MA). Of the remaining filtrate, 300 µl was spun down at 5000*g* for 3 min. The supernatant was removed and incubated with CellROX for 2 h at 25°C, and fluorescence measurements were conducted as described previously. The resulting measurements were normalized against total cell counts.

### (k) Inter-strain comparisons of oxidative stress-related genes

To compare antioxidative responses of the three studied strains, differential expression analyses were carried out between strains at the same temperature in a pairwise fashion (i.e. H2 versus CC7, RS versus CC7, RS versus H2). This was possible because our analyses were based on data mapped against *Aiptasia* gene models [26] instead of against *de novo* strain-specific transcriptomes. Methods used to carry out these analyses were analogous to the comparisons of transcriptomes and proteomes of the same strain under different temperatures described in previous subsections.

From the resulting outputs, we specifically searched for 98 gene models annotated with putative oxidative stress-related function (e.g. thioredoxin reductase, superoxide dismutase, glutathione S-transferase and peroxidasin). The outputs were further concatenated into tables that list the relative fold changes of all relevant pairwise comparisons (electronic supplementary material, supplemental dataset S2).

### (l) Typing of the ITS2 regions of Red Sea symbionts

Extracted DNA from four RS *Aiptasia* at 25°C were subjected to a PCR targeting the ITS2 regions of *Symbiodinium* (using the primers SYM_VAR_FWD and SYM_VAR_REV [35]). These samples were 4 of 96 samples sequenced on a MiSeq run. Reads from these samples were pooled (757 132 paired-end reads), trimmed with cutadapt v1.16 [36], and merged into a single read with bbmerge v37.93 [37] to produce 348 488 reads (with length ≥200 bp).

These reads were clustered at 97% similarity with cdhit-est v4.7 [38] to produce 73 clusters—the 10 most abundant clusters were parsed with a Python script. Representative sequences of these clusters were subjected to a BLASTN search against nt, and clade information of the best *Symbiodinium* hits were searched for manually.

## 3. Results

### (a) Weak correlation between transcript and protein levels

**Figure 2.**
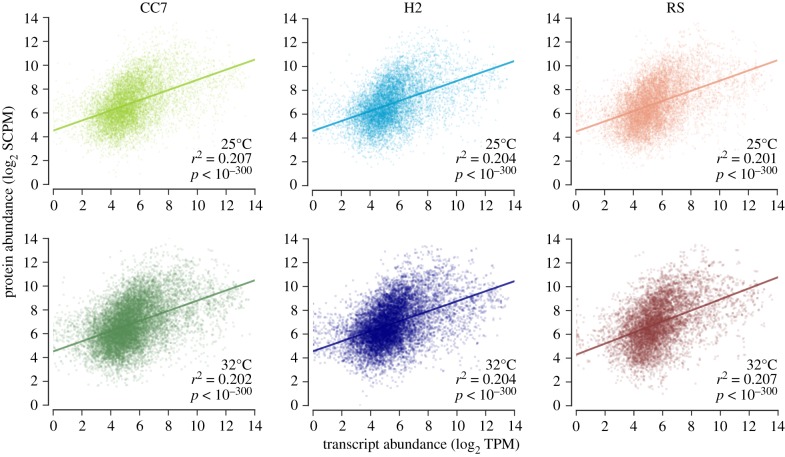
Correlation of protein abundance with mRNA expression at control (25°C) and heat stress (32°C) for each biological replicate. Expression values were log_2_-transformed to better approximate normal distributions. Linear regressions were calculated for control and stress conditions across all replicates of a given strain. Darker colours represent heat stress; lighter ones represent control. SCPM, spectral counts per million; TPM, transcripts per million.

Similar to previous studies that combined transcriptomic and proteomic approaches, we found substantially more genes with detectable transcripts (23 635 genes, 85.9% of all *Aiptasia* gene models) than proteins (4014 genes, 14.6%). Of the total detected proteins, 98% were successfully assigned to a matching transcript. To assess correlations between transcript and protein abundances, we performed linear regressions for samples at control (25°C) and under heat stress (32°C) (figure 2). These analyses indicated a similarly weak but highly significant correlation (*r*^2^ ≈ 0.2, *p*-value < 10^−300^) across all strains and temperatures, suggesting that short-term heat stress does not significantly alter the correlation between absolute transcript and protein levels.

Furthermore, we investigated whether transcripts and their respective proteins display similar relative changes under thermal stress. Surprisingly, the previously identified moderate correlation was abolished when correlating the temperature-driven changes of transcripts to that of their proteins (all *r*^2^ ≃ 0; electronic supplementary material, figure S1). We hypothesized that the poor correlation of temperature-driven changes could be owing to a large proportion of proteins not being significantly different between the tested conditions (mean = 6%). It is possible that proteins with significant fold change would have transcripts differentially expressed in the same direction—thus, the removal of all non-significantly expressed proteins should result in an improved correlation. However, the correlation remained zero across all strains (electronic supplementary material, figure S2, *r*^2^ ≃ 0), indicating that significant changes of protein abundances occur independently from detectable changes in transcript levels.

To better understand this paradox, we investigated the direction of expression change. Focusing on the subset of differentially expressed proteins, we observed roughly equal numbers of concordant changes (e.g. upregulated protein and upregulated transcript) and discordant ones (e.g. upregulated protein with downregulated transcript) (electronic supplementary material, figure S3). Overall, RS had the highest concordance (12%), followed by CC7 (7%) and H2 (3%). The lack of protein–mRNA matches, along with the presence of oppositely matching pairs, provide an explanation for the previously observed absence in fold change correlations.

### (b) Inter-strain transcriptome and proteome heat stress responses

To assess inter-strain response to heat stress, we compared the lists of differentially expressed transcripts (DETs) and proteins (DEPs). Initial analysis revealed H2 as an outlier, with only 510 DETs detected in the transcriptome in comparison to 1226 and 2888 in CC7 and RS respectively. Comparison of DETs between strains showed 170 common genes (83 up, 87 downregulated). Included in these are established heat response genes such as HSP90, HSP70, HSP90 co-chaperone Cdc37, glutathione peroxidase, superoxide dismutase, calreticulin and calumenin-B. While CC7 and H2 had around 50% of their total DETs downregulated, RS had 57%. Additionally, 67% of RS DETs were not detected in any of the other strains (electronic supplementary material, table S2). In particular, we found more HSP70, superoxide dismutase (SOD), glutathione S-transferase and peroxidasin transcripts differently expressed in RS than in the other strains. Comparison of DETs between strains showed that RS and CC7 (613 DETs) had more similarities to each other than to H2 (RS versus H2: 326, CC7 versus H2: 185).

The number of DEPs after heat stress was generally much lower, with a maximum of 294 proteins in CC7, followed by 275 and 151 in H2 and RS respectively showing absolute fold-changes of more than 1.2. Using a *t*-test, only one protein was deemed significantly expressed after multiple test correction (corrected *p* < 0.05): AIPGENE10033 (ADP ribosylation-related protein) in RS. Similarly, a GLM as employed by Oakley, Durand [32] identified only one protein that was differentially expressed due to temperature: AIPGENE18140 (programmed cell death interacting protein).

### (c) Functional enrichment of differentially expressed transcripts and proteins

Functional enrichment of the DETs revealed a total of 24 significantly enriched GO terms that overlapped between the strains (electronic supplementary material, table S3). Particular noteworthy terms were related to protein misfolding, oxidative stress, mRNA splicing, endoplasmic reticulum stress and iron binding.

**Figure 3.**
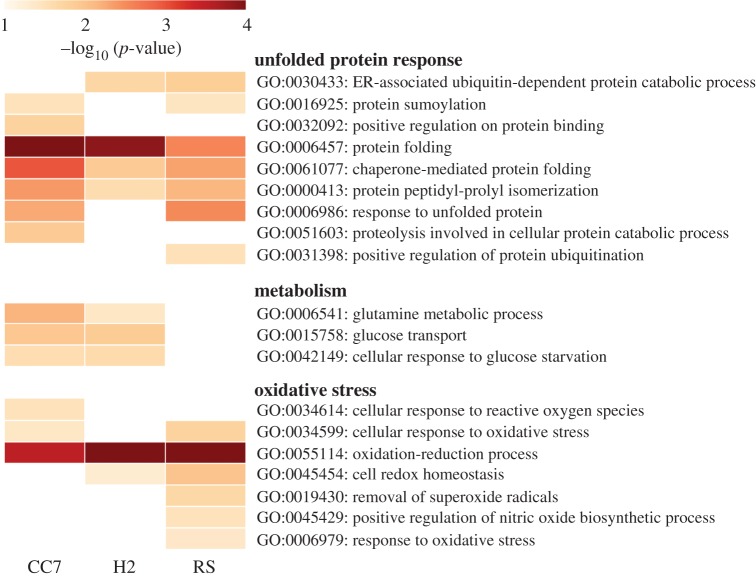
Heatmap of GO terms related to unfolded protein response, metabolism and oxidative stress from three *Aiptasia pallida* transcriptomes. Selected terms illustrate strain-specific differential transcriptomic responses to heat stress (*p* < 0.05). Empty boxes denote differences that were not significant (*p* ≥ 0.05).

When categorized into higher-order terms, protein misfolding pathways appear to be positively enriched in all strains, especially CC7 (electronic supplementary material, supplemental dataset S1). In addition, terms related to oxidative stress response were predominant in RS, showing twice as many than any of the other strains (figure 3). Included in these were unique responses related to superoxide dismutase (GO:0004784), regulation of nitric oxide (GO:0045429) and peroxidase activity (GO:0004601). H2 lacked many of the coping pathway enrichments seen in the other strains (figure 3). As there were few DEPs that passed our stringent analysis, we were unable to perform a similar functional enrichment analysis for the protein data.

Overall, RS and CC7 both showed a broader response to heat stress than H2, through their activation of a variety of gene homologues and essential stress response pathways; indicating strain-specific differences in temperature response. However, both DET and GO-term enrichment analysis indicated a strong antioxidant response in RS anemones, which stood apart from the others (electronic supplementary material, tables S2 and S3; figure 3). Hence, we hypothesized that RS anemones may have adapted to warmer waters through improved antioxidant capacity. Additionally, while our investigation centred around the role of the cnidarian host, we kept under consideration that the symbiont's thermal tolerance may be contributing to the oxidative stress experience and response of the host. Previous studies have genotyped CC7 and H2 associated symbionts as clade A (subclade A4) and B (subclade B1) respectively. Similarly, RS anemones associate with a mix of clades A and B *Symbiodinium* (subclades A1, A2, A4 and B1) (electronic supplementary material, table S1). To validate our hypothesis, we conducted a biochemical ROS assay.

### (d) Biochemical quantification of reactive oxygen species

**Figure 4.**
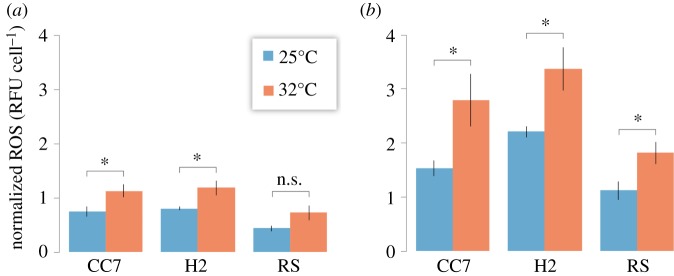
ROS produced by *Symbiodinium* (*a*) *in hospite* and (*b*) in isolate. ROS was measured for control (25°C) and heat stress (32°C) conditions and normalized against total symbiont counts. Error bars denote ±1 standard errors. *p < 0.05; n.s., not significant.

Based on the transcriptomic responses, we predicted differences in the ability of the different host genotypes in mitigating higher ROS levels in response to heat stress. In order to assess if these differences were indeed manifested on the physiological level and whether they are host- or symbiont-driven, we quantified ROS levels of the three *Aiptasia* hosts by first assaying ROS production *in hospite* (i.e. host and symbiont) in response to heat stress (figure 4*a*). We did not detect any significant changes in symbiont densities in any of the anemone strains (electronic supplementary material, table S4). Subsequently, we measured the ROS production of freshly isolated *Symbiodinium* cultures from the respective host (i.e. symbiont only) in response to the same stress (figure 4*b*).

ROS abundance was generally lowest in RS and there was no significant increase in ROS production in RS under heat stress (*p* = 0.093) unlike in CC7 and H2 (*p* < 0.05 both) (figure 4*a*). However, when we compared these ROS levels to those of isolated symbionts we found that the ROS levels measured *in hospite* mirrored those of the freshly isolated symbionts (figure 4*b*). For all strains and conditions measured we observed a generally lower level of ROS *in hospite* than in the isolated symbionts, emphasizing the ability of the host in mitigating some of the ROS produced. Nonetheless, the ROS production pattern appeared to be predominantly driven by the symbiont.

### (e) Inter-strain comparisons of oxidative stress-related genes

We were curious to what extent ROS levels were dependent on the antioxidant capacity of the host, or the overall production of the symbiont. A possible confounding factor of the ROS assay was that the different strains of *Aiptasia* could have had different initial expressions of oxidative stress-related genes. Thus, the lower amounts of ROS assayed in RS samples could potentially be attributed to a higher initial level of anti-ROS transcripts in the host, and not owing to its symbiont being more adapted to heat stress. To de-convolute the role of the host further, we performed inter-strain transcriptomic and proteomic comparisons, focusing on a set of 98 genes with oxidative stress-related functions (electronic supplementary material, supplemental dataset S2).

We found that CC7 consistently had a higher abundance in antioxidant transcripts under control conditions compared to RS and H2 (24/20 and 17/12 respectively). When exposed to temperature stress, CC7 had even more transcripts at significantly higher abundances compared to both other strains. However, on a protein level, we could not detect convincing differences in the abundances of antioxidants in relation to their condition. Proteins relating to glutathione S-transferase, SOD and thioredoxin showed an overall trend of being more abundant in CC7 and H2 under control conditions. CC7 maintained significantly higher abundances of thioredoxin and SOD proteins, particularly when compared to RS. Thus, the analysis showed that: (i) RS did not have a higher antioxidant capacity than other strains; (ii) initial higher antioxidant response was not the driver of the ROS abundance measured in the assay; and (iii) oxidative stress experienced by the host is therefore mostly determined by the associated symbiont and not the antioxidant capacity of the host.

## 4. Discussion

### (a) Transcriptome-proteome correlations in a cnidarian model organism

The weak but significant correlation (*r*^2^ ≈ 0.2, figure 2) between the transcriptome and proteome observed across all three investigated *Aiptasia* strains echo similar results in other model organisms, such as in yeast (*r*^2^ = 0.66) [39] and in mice (*r*^2^ = 0.34) [40,41]. Correlation of fold changes in protein and transcripts in response to heat stress were completely absent (*r*^2^ ≃ 0; electronic supplementary material, figure S1), again similar to observations in other model organisms [22,42,43]. While technical limitations can play a crucial factor [44], this independence is primarily biological in nature. The exposure to stress is one of various factors that can cause uncoupling of transcription and translation as a tool of biological fine-tuning (see review by Maier, Guell [40]). Since proteome changes are naturally time-dependent, and in light of protein misfolding owing to heat stress probably further delayed [45], we cannot exclude time-lag as a potential factor for the absence in fold change correlation in this study. Our observations on transcript and protein correlations are therefore not surprising, but a valuable addition to the limited number of correlation studies and the first in cnidarians. In agreement with other studies [46], results suggest that transcripts alone might not be sufficient to deduce organismal proteomic responses to stress.

### (b) Strain-specific responses to heat stress

Comparison of inter-strain heat stress responses highlighted several common pathways that suggest a core cnidarian response mechanism. This core response includes regulation of protein folding, oxidative stress response and calcium homeostasis [14,47]. Additionally, we observed splicing-related categories among the strains. This is not surprising, as heat shock has been shown to repress mRNA splicing in eukaryotic cells [48] and similar impacts were recently reported in corals [49]. Repressed splicing can act as a form of controlled protein production, allowing for selective expression of stress adaptation related proteins [50]. Sophisticated forms of transcriptional regulation therefore appear to play a more crucial role in cnidarian stress response than so far accredited. These processes might also contribute to the observed lack of correlation between transcriptome and proteome.

The extensive transcriptional regulation of RS anemones stands apart from that of CC7 and H2. The significantly higher number of DETs observed in RS, of which most are exclusive, may be a form of expression plasticity allowing for a faster stress response [51]. This potentially sophisticated form of gene expression control could be crucial to short- and long-term adaptations [13,52]. The higher proportion of downregulated genes may indicate that RS is able to maintain metabolic activity at reduced levels (figure 3). Control and stability of the metabolism during exposure to stressors has previously been shown to increase survival time of organisms [53] and hence plays a crucial role in heat shock resilience.

On a gene-family level, differences were not only obvious in terms of presence or absence, but also in the number of gene variants expressed. This held particularly true for RS and CC7, where both usually expressed crucial stress response genes but RS used various gene variants rather than only selected ones (electronic supplementary material, table S2). An example of such is the more abundant expression of HSP70 in RS, followed by CC7 and an absence in H2. As expression of HSP70 has shown to be a distinguishing factor between thermally tolerant and less tolerant corals [54], the results suggest that RS has an improved capacity in initial response to heat stress and has a higher thermal tolerance. This would be expected based on the significantly higher average summer temperatures prevalent in the RS (figure 1). Similar patterns were observed in genes related to oxidative stress response. Noteworthy is the significant upregulation of Nrf2 (nuclear factor erythroid-2) observed only in RS (electronic supplementary material, table S2). Nrf2 has emerged as an important regulator of cellular resistance to oxidants and controls an array of downward antioxidant responses [55]. Absence of antioxidant response in the cnidarian proteome during a 2 day heat stress was previously observed in *Acropora micropthalma* [31]. Instead, the strongest proteome antioxidant response was detected in the associated symbionts, indicating their role in controlling host oxidative stress.

### (c) Oxidative stress response is dependent on the thermotolerance of associated symbionts

Efficient control of ROS has long been a mechanism accredited to thermal tolerant corals [4]. However, the current perspective on oxidative stress resilience is a mixed one where both partners, the coral host and the symbiont, have shown to contribute [17,18]. Since thermotolerance of *Symbiodinium* is highly variable, knowing the associate symbiont clades is important in understanding holobiont thermal resilience; clade A *Symbiodinium* are generally consider more thermotolerant [56,57].

Our multi-omics approach showed that although all strains shift expression of antioxidant genes, RS appeared to show the highest antioxidant capacity. However, physiological measurements showed that the ROS level exposure of the host was predominantly driven by the symbiont. Interestingly, while the host evidently showed the ability to mitigate ROS produced by the symbiont, inter-strain comparisons suggest that the mitigation capacity of CC7, H2 and RS were fairly similar.

Consistent with patterns observed throughout this study, RS and CC7 had higher number of antioxidant-related transcripts than H2 in either condition. Nonetheless, we show that RS does not express significantly more antioxidant genes or proteins than the other two strains. Indeed, we detected the strongest presence of antioxidant genes in CC7 under control and heat stress. On a protein level, the most drastic changes in abundance were also observed in CC7, undermining the possibility of antioxidant capacity as an RS-specific adaptation to heat stress. Thus, there was no indication that the observed difference in ROS was driven by genotype response capacity. This corroborates that the thermal tolerance of associated symbionts is the main determinant of ROS levels and hence the overall oxidative stress experienced by the host [6,58]. The ability to establish associations with symbionts that have a higher thermal tolerance may be the most crucial mechanism underlying the adaptation of zooxanthellate cnidarians to increased temperature environments. Previous studies on *Symbiodinium* associated with RS corals have shown higher thermotolerance that may effectively translate into higher tolerance for the holobiont in total [35,59]. Our findings presented here support the notion that RS cnidarians adapted to their warmer surroundings through symbiosis with local, thermotolerant symbionts, instead of increasing their antioxidative capacity to deal with the increased ROS generated by less tolerant symbionts.

There is enormous interest in understanding the drivers of thermal resilience and susceptibility in corals. Results presented here highlight the complexity and interacting contribution of both partners. Efficient transcriptional regulation and plasticity appear to be important coping mechanisms of pre-exposed cnidarians and are probably important for the initial response to increasing temperatures. However, not all responses observed on a transcriptomic level translated into predicted proteomic or physiological results. Our multi-omics approach highlights the importance of secondary validation in order to produce confident and meaningful biological conclusions. With regards to oxidative stress specifically, physiological measurements validate our hypothesis that the symbiont exerts a significant influence on the overall ROS level within the host. Colonization of extreme environments may therefore have been possible through associations with more thermotolerant symbionts. However, this relation may be evolutionarily conserved, suggesting that the infection of other genotypes with heat-tolerant symbionts may not necessarily lead to overall improved tolerance. The findings presented here emphasize the importance of the symbiont in determining the thermal resilience of the holobiont, a crucial factor to consider for future attempts in aiding corals through predicted climate change impacts.

## Supplementary Material

Electronic supplementary materials

## Supplementary Material

Supplemental Dataset S1

## Supplementary Material

Supplemental Dataset S2

## Data accessibility

RNA-seq data are available at NCBI under project number PRJNA406873. Python scripts for proteomic analysis, and the Excel sheet providing normalized protein intensities, are available at https://github.com/lyijin/aiptasia_multi-omics, with more detailed explanations.

## References

[1] Fisher R, O'Leary RA, Low-Choy S, Mengersen K, Knowlton N, Brainard RE, Caley MJ 2015 Species richness on coral reefs and the pursuit of convergent global estimates. Curr. Biol. 25, 500–505. (10.1016/j.cub.2014.12.022) 25639239

[2] Hoegh-Guldberg O 1999 Climate change, coral bleaching and the future of the world's coral reefs. Mar. Freshw. Res. 50, 839.

[3] Brown BE 1997 Coral bleaching: causes and consequences. Coral Reefs 16, S129–S138. (10.1007/s003380050249)

[4] Barshis DJ, Ladner JT, Oliver TA, Seneca FO, Traylor-Knowles N, Palumbi SR 2013 Genomic basis for coral resilience to climate change. Proc. Natl Acad. Sci. USA 110, 1387–1392. (10.1073/pnas.1210224110) 23297204 PMC3557039

[5] Schoepf V, Stat M, Falter JL, McCulloch MT 2015 Limits to the thermal tolerance of corals adapted to a highly fluctuating, naturally extreme temperature environment. Sci. Rep. 5, 17639 (10.1038/srep17639) 26627576 PMC4667274

[6] Leggat W, Seneca F, Wasmund K, Ukani L, Yellowlees D, Ainsworth TD 2011 Differential responses of the coral host and their algal symbiont to thermal stress. PLoS ONE 6, e26687 (10.1371/journal.pone.0026687) 22039532 PMC3200360

[7] Pinzon JH, Kamel B, Burge CA, Harvell CD, Medina M, Weil E, Mydlarz LD 2015 Whole transcriptome analysis reveals changes in expression of immune-related genes during and after bleaching in a reef-building coral. R. Soc. open sci. 2, 140214 (10.1098/rsos.140214) 26064625 PMC4448857

[8] Tong H, Cai L, Zhou G, Yuan T, Zhang W, Tian R, Huang H, Qian PY 2017 Temperature shapes coral-algal symbiosis in the South China Sea. Sci. Rep. 7, 40118 (10.1038/srep40118) 28084322 PMC5234030

[9] Silverstein RN, Cunning R, Baker AC 2015 Change in algal symbiont communities after bleaching, not prior heat exposure, increases heat tolerance of reef corals. Glob. Chang Biol. 21, 236–249. (10.1111/gcb.12706) 25099991

[10] Boulotte NM, Dalton SJ, Carroll AG, Harrison PL, Putnam HM, Peplow LM, van Oppen MJ 2016 Exploring the *Symbiodinium* rare biosphere provides evidence for symbiont switching in reef-building corals. ISME J. 10, 2693–2701. (10.1038/ismej.2016.54) 27093048 PMC5113844

[11] Hume BC, Voolstra CR, Arif C, D'Angelo C, Burt JA, Eyal G, Loya Y, Wiedenmann J 2016 Ancestral genetic diversity associated with the rapid spread of stress-tolerant coral symbionts in response to Holocene climate change. Proc. Natl Acad. Sci. USA 113, 4416–4421. (10.1073/pnas.1601910113) 27044109 PMC4843444

[12] van Oppen MJ, Oliver JK, Putnam HM, Gates RD 2015 Building coral reef resilience through assisted evolution. Proc. Natl Acad. Sci. USA 112, 2307–2313. (10.1073/pnas.1422301112) 25646461 PMC4345611

[13] Kenkel CD, Matz MV 2016 Gene expression plasticity as a mechanism of coral adaptation to a variable environment. Nat. Ecol. Evol. 1, 14 (10.1038/s41559-016-0014) 28812568

[14] DeSalvo MK, Sunagawa S, Voolstra CR, Medina M 2010 Transcriptomic responses to heat stress and bleaching in the elkhorn coral *Acropora palmata*. Mar. Ecol. Prog. Ser. 402, 97–113. (10.3354/meps08372)

[15] Weis VM 2008 Cellular mechanisms of Cnidarian bleaching: stress causes the collapse of symbiosis. J. Exp. Biol. 211, 3059–3066. (10.1242/jeb.009597) 18805804

[16] Tchernov D, Kvitt H, Haramaty L, Bibby TS, Gorbunov MY, Rosenfeld H, Falkowski PG 2011 Apoptosis and the selective survival of host animals following thermal bleaching in zooxanthellate corals. Proc. Natl Acad. Sci. USA 108, 9905–9909. (10.1073/pnas.1106924108) 21636790 PMC3116386

[17] Howells EJ, Beltran VH, Larsen NW, Bay LK, Willis BL, van Oppen MJH 2011 Coral thermal tolerance shaped by local adaptation of photosymbionts. Nat. Clim. Chang 2, 116–120. (10.1038/nclimate1330)

[18] Cunning R, Baker AC 2012 Excess algal symbionts increase the susceptibility of reef corals to bleaching. Nat. Clim. Chang. 3, 259–262. (10.1038/nclimate1711)

[19] Drake JL, Mass T, Haramaty L, Zelzion E, Bhattacharya D, Falkowski PG 2013 Proteomic analysis of skeletal organic matrix from the stony coral *Stylophora pistillata*. Proc. Natl Acad. Sci. USA 110, 3788–3793. (10.1073/pnas.1301419110) 23431140 PMC3593878

[20] Ramos-Silva P, Kaandorp J, Huisman L, Marie B, Zanella-Cleon I, Guichard N, Miller DJ, Marin F 2013 The skeletal proteome of the coral *Acropora millepora*: the evolution of calcification by co-option and domain shuffling. Mol. Biol. Evol. 30, 2099–2112. (10.1093/molbev/mst109) 23765379 PMC3748352

[21] Nie L, Wu G, Zhang W 2006 Correlation of mRNA expression and protein abundance affected by multiple sequence features related to translational efficiency in *Desulfovibrio vulgaris*: a quantitative analysis. Genetics 174, 2229–2243. (10.1534/genetics.106.065862) 17028312 PMC1698625

[22] Vogel C, Silva GM, Marcotte EM 2011 Protein expression regulation under oxidative stress. Mol. Cell. Proteomics 10, M111.009217 (10.1074/mcp.M111.009217) PMC3237073 21933953

[23] Grajales A, Rodriguez E 2014 Morphological revision of the genus *Aiptasia* and the family Aiptasiidae (Cnidaria, Actiniaria, Metridioidea). Zootaxa 3826, 55–100. (10.11646/zootaxa.3826.1.2) 24990039

[24] Sunagawa S, Wilson EC, Thaler M, Smith ML, Caruso C, Pringle JR, Weis VM, Medina M, Schwarz JA 2009 Generation and analysis of transcriptomic resources for a model system on the rise: the sea anemone *Aiptasia pallida* and its dinoflagellate endosymbiont. BMC Genomics 10, 258 (10.1186/1471-2164-10-258) 19500365 PMC2702317

[25] Xiang T, Hambleton EA, DeNofrio JC, Pringle JR, Grossman A 2013 Isolation of clonal axenic strains of the symbiotic dinoflagellate *Symbiodinium* and their growth and host specificity. J. Phycol. 49, 447–458. 27007034 10.1111/jpy.12055

[26] Baumgarten S et al. 2015 The genome of *Aiptasia*, a sea anemone model for coral symbiosis. Proc. Natl Acad. Sci. USA 112, 11 893–11 898. (10.1073/pnas.1513318112) PMC4586855 26324906

[27] Bray NL, Pimentel H, Melsted P, Pachter L 2016 Near-optimal probabilistic RNA-seq quantification. Nat. Biotechnol. 34, 525–527. 27043002 10.1038/nbt.3519

[28] Pimentel H, Bray NL, Puente S, Melsted P, Pachter L 2017 Differential analysis of RNA-seq incorporating quantification uncertainty. Nat. Methods 14, 687–690. (10.1038/nmeth.4324) 28581496

[29] Cox J, Mann M 2008 MaxQuant enables high peptide identification rates, individualized p.p.b.-range mass accuracies and proteome-wide protein quantification. Nat. Biotechnol. 26, 1367–1372. (10.1038/nbt.1511) 19029910

[30] Herbrich SM et al. 2013 Statistical inference from multiple iTRAQ experiments without using common reference standards. J. Proteome Res. 12, 594–604. (10.1021/pr300624g) 23270375 PMC4223774

[31] Weston AJ, Dunlap WC, Beltran VH, Starcevic A, Hranueli D, Ward M, Long PF 2015 Proteomics links the redox state to calcium signaling during bleaching of the scleractinian coral *Acropora microphthalma* on exposure to high solar irradiance and thermal stress. Mol. Cell. Proteomics 14, 585–595. (10.1074/mcp.M114.043125) 25561505 PMC4349979

[32] Oakley CA, Durand E, Wilkinson SP, Peng L, Weis VM, Grossman AR, Davy SK 2017 Thermal shock induces host proteostasis disruption and endoplasmic reticulum stress in the model symbiotic Cnidarian *Aiptasia*. J. Proteome Res. 16, 2121–2134. (10.1021/acs.jproteome.6b00797) 28474894

[33] Benjamini Y, Yekutieli D 2001 The control of the false discovery rate in multiple testing under dependency. Ann. Stat. 29, 1165–1188.

[34] Alexa A, Rahnenfuhrer J, Lengauer T 2006 Improved scoring of functional groups from gene expression data by decorrelating GO graph structure. Bioinformatics 22, 1600–1607. (10.1093/bioinformatics/btl140) 16606683

[35] Hume B, D'Angelo C, Burt J, Baker AC, Riegl B, Wiedenmann J 2013 Corals from the Persian/Arabian Gulf as models for thermotolerant reef-builders: prevalence of clade C3 *Symbiodinium*, host fluorescence and *ex situ* temperature tolerance. Mar. Pollut. Bull. 72, 313–322. (10.1016/j.marpolbul.2012.11.032) 23352079

[36] Martin M 2011 Cutadapt removes adapter sequences from high-throughput sequencing reads. EMBnet J. 17, 10–12.

[37] Bushnell B, Rood J, Singer E 2017 BBMerge—Accurate paired shotgun read merging via overlap. PLoS ONE 12, e0185056 (10.1371/journal.pone.0185056) 29073143 PMC5657622

[38] Li W, Godzik A 2006 Cd-hit: a fast program for clustering and comparing large sets of protein or nucleotide sequences. Bioinformatics 22, 1658–1659. (10.1093/bioinformatics/btl158) 16731699

[39] Greenbaum D, Colangelo C, Williams K, Gerstein M 2003 Comparing protein abundance and mRNA expression levels on a genomic scale. Genome Biol. 4, 117 (10.1186/gb-2003-4-9-117) 12952525 PMC193646

[40] Maier T, Guell M, Serrano L 2009 Correlation of mRNA and protein in complex biological samples. FEBS Lett. 583, 3966–3973. (10.1016/j.febslet.2009.10.036) 19850042

[41] Vogel C, Marcotte EM 2012 Insights into the regulation of protein abundance from proteomic and transcriptomic analyses. Nat. Rev. Genet. 13, 227–232. (10.1038/nrg3185) 22411467 PMC3654667

[42] Lee MV, Topper SE, Hubler SL, Hose J, Wenger CD, Coon JJ, Gasch AP 2011 A dynamic model of proteome changes reveals new roles for transcript alteration in yeast. Mol. Syst. Biol. 7, 514 (10.1038/msb.2011.48) 21772262 PMC3159980

[43] Cheng Z, Teo G, Krueger S, Rock TM, Koh HW, Choi H, Vogel C 2016 Differential dynamics of the mammalian mRNA and protein expression response to misfolding stress. Mol. Syst. Biol. 12, 855 (10.15252/msb.20156423) 26792871 PMC4731011

[44] Samaj J, Thelen JJ 2007 Plant proteomics, 368 p Berlin, Germany: Spinger-Verlag Berlin Heidelberg.

[45] Gidalevitz T, Prahlad V, Morimoto RI 2011 The stress of protein misfolding: from single cells to multicellular organisms. Cold Spring Harb. Perspect. Biol. 3, 1–18. (10.1101/cshperspect.a009704) PMC3098679 21536706

[46] Liu Y, Beyer A, Aebersold R 2016 On the dependency of cellular protein levels on mRNA abundance. Cell 165, 535–550. (10.1016/j.cell.2016.03.014) 27104977

[47] Maor-Landaw K, Levy O 2016 Gene expression profiles during short-term heat stress; branching vs. massive Scleractinian corals of the Red Sea. PeerJ 4, e1814 (10.7717/peerj.1814) 27069783 PMC4824894

[48] Yamamoto K, Furukawa MT, Fukumura K, Kawamura A, Yamada T, Suzuki H, Hirose T, Sakamoto H, Inoue K 2016 Control of the heat stress-induced alternative splicing of a subset of genes by hnRNP K. Genes Cells 21, 1006–1014. (10.1111/gtc.12400) 27491955

[49] Seneca FO, Palumbi SR 2015 The role of transcriptome resilience in resistance of corals to bleaching. Mol. Ecol. 24, 1467–1484. (10.1111/mec.13125) 25728233

[50] Hartl FU, Bracher A, Hayer-Hartl M 2011 Molecular chaperones in protein folding and proteostasis. Nature 475, 324–332. (10.1038/nature10317) 21776078

[51] Brown BE, Downs CA, Dunne RP, Gibb SW 2002 Exploring the basis of thermotolerance in the reef coral *Goniastrea aspera*. Mar. Ecol. Prog. Ser. 242, 119–129.

[52] de Nadal E, Ammerer G, Posas F 2011 Controlling gene expression in response to stress. Nat. Rev. Genet. 12, 833–845. (10.1038/nrg3055) 22048664

[53] Hand SC, Hardewig I 1996 Downregulation of cellular metabolism during environmental stress: mechanisms and implications. Annu. Rev. Physiol. 58, 539–563. (10.1146/annurev.ph.58.030196.002543) 8815808

[54] Bellantuono AJ, Hoegh-Guldberg O, Rodriguez-Lanetty M 2012 Resistance to thermal stress in corals without changes in symbiont composition. Proc. Biol. Sci. 279, 1100–1107. (10.1098/rspb.2011.1780) 21976690 PMC3267153

[55] Nguyen T, Nioi P, Pickett CB 2009 The Nrf2-antioxidant response element signaling pathway and its activation by oxidative stress. J. Biol. Chem. 284, 13 291–13 295. (10.1074/jbc.R900010200) PMC2679427 19182219

[56] Robison JD, Warner ME 2006 Differential impacts of photoacclimation and thermal stress on the photobiology of four different phylotypes of *Symbiodinium* (Pyrrhophyta). J. Phycol. 42, 568–579. (10.1111/j.1529-8817.2006.00232.x)

[57] Warner ME, Suggett DJ 2016 The photobiology of *Symbiodinium* spp.: linking physiological diversity to the implications of stress and resilience. In The Cnidaria, past, present and future (eds Goffredo S, Dubinsky Z), pp. 489–509. Berlin, Germany: Springer.

[58] Roth MS 2014 The engine of the reef: photobiology of the coral-algal symbiosis. Front. Microbiol. 5, 422 (10.3389/fmicb.2014.00422) 25202301 PMC4141621

[59] Ziegler M, Roder C, Buchel C, Voolstra CR 2015 Niche acclimatization in Red Sea corals is dependent on flexibility of host-symbiont association. Mar. Ecol. Prog. Ser. 533, 149–161. (10.3354/meps11365)

