# Supplementary material for: Multi-omics analysis of thermal stress response in a zooxanthellate cnidarian reveals the importance of associating with thermotolerant symbionts
Source: Proc Biol Sci. 2018 Apr 18;285(1877):20172654. doi: 10.1098/rspb.2017.2654 (PMC5936724; doi:10.1098/rspb.2017.2654)
Supplement: Electronic supplementary materials [file rspb20172654supp1.docx]

**Supplementary Figures and Tables: Multi-omics analysis of thermal stress response in a zooxanthellate cnidarian reveals the importance of associating with thermotolerant symbionts**

Maha J. Cziesielski*^1^, Yi Jin Liew*^1^, Guoxin Cui^1^, Sebastian Schmidt-Roach^1^, Sara Campana^1^, Claudius Marondedze^1@^ and Manuel Aranda^1^#

^1^King Abdullah University of Science and Technology (KAUST), Red Sea Research Center (RSRC), Biological and Environmental Sciences & Engineering Division (BESE), Thuwal, Saudi Arabia

@Current address: Laboratoire de Physiologie Cellulaire et Végétale, Université Grenoble Alpes, CEA/BIG, 17 avenue des Martyrs, 38054 Grenoble, France

#Correspondence: Manuel Aranda, E-mail: manuel.aranda@kaust.edu.sa

*These authors contributed equally.

**
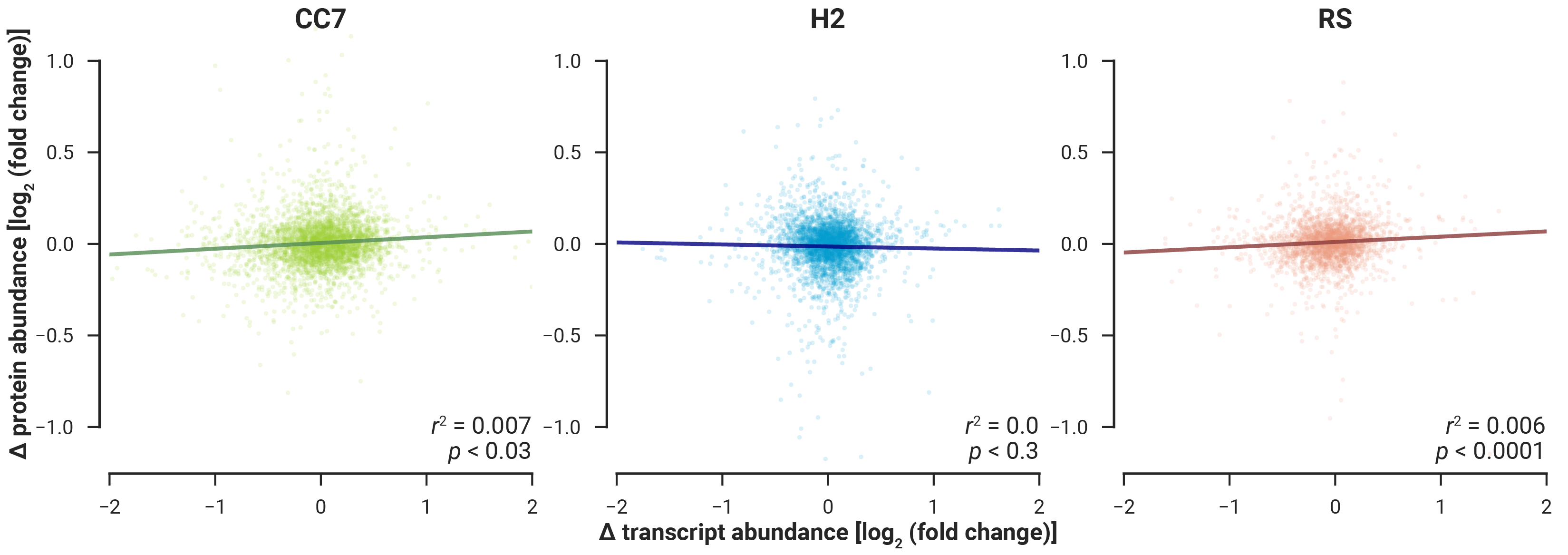
**

**Figure S1. Linear correlations of temperature-driven changes in mRNA expression and protein expression.** All detected proteins were considered and matched with their respective transcripts. Fold changes were calculated as expression level under heat stress divided by expression at control, then log_2_-transformed to better approximate normal distributions. Darker lines represent linear regressions for the respective strains.

**
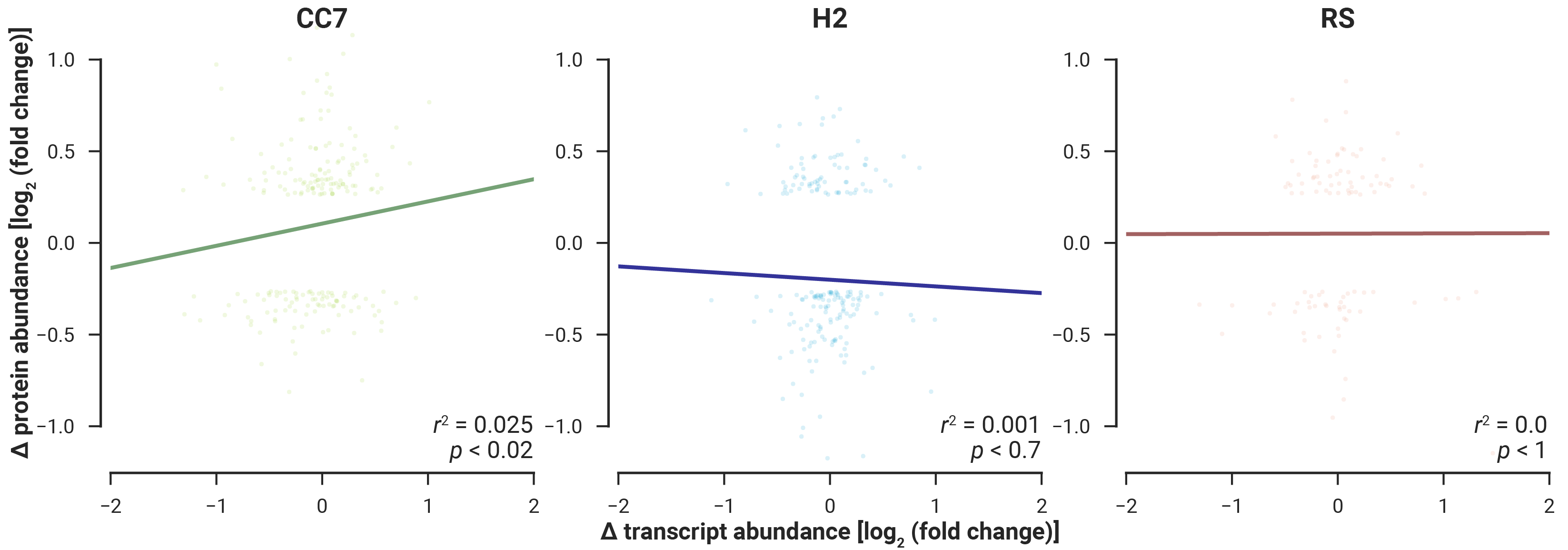
**

**Figure S2: Linear correlations of temperature-driven changes in significantly expressed proteins and matching mRNA.** Only significantly expressed proteins were considered to test whether according transcripts showed similar significant changes. Fold changes were calculated as expression level under heat stress divided by expression at control, then log_2_-transformed to better approximate normal distributions. Except for CC7, none of the strains showed a positive correlation between significant changes in protein and mRNA. Although CC7 correlations were positive, values were low and did not indicate an improvement to the previous analysis where all proteins were considered.

**
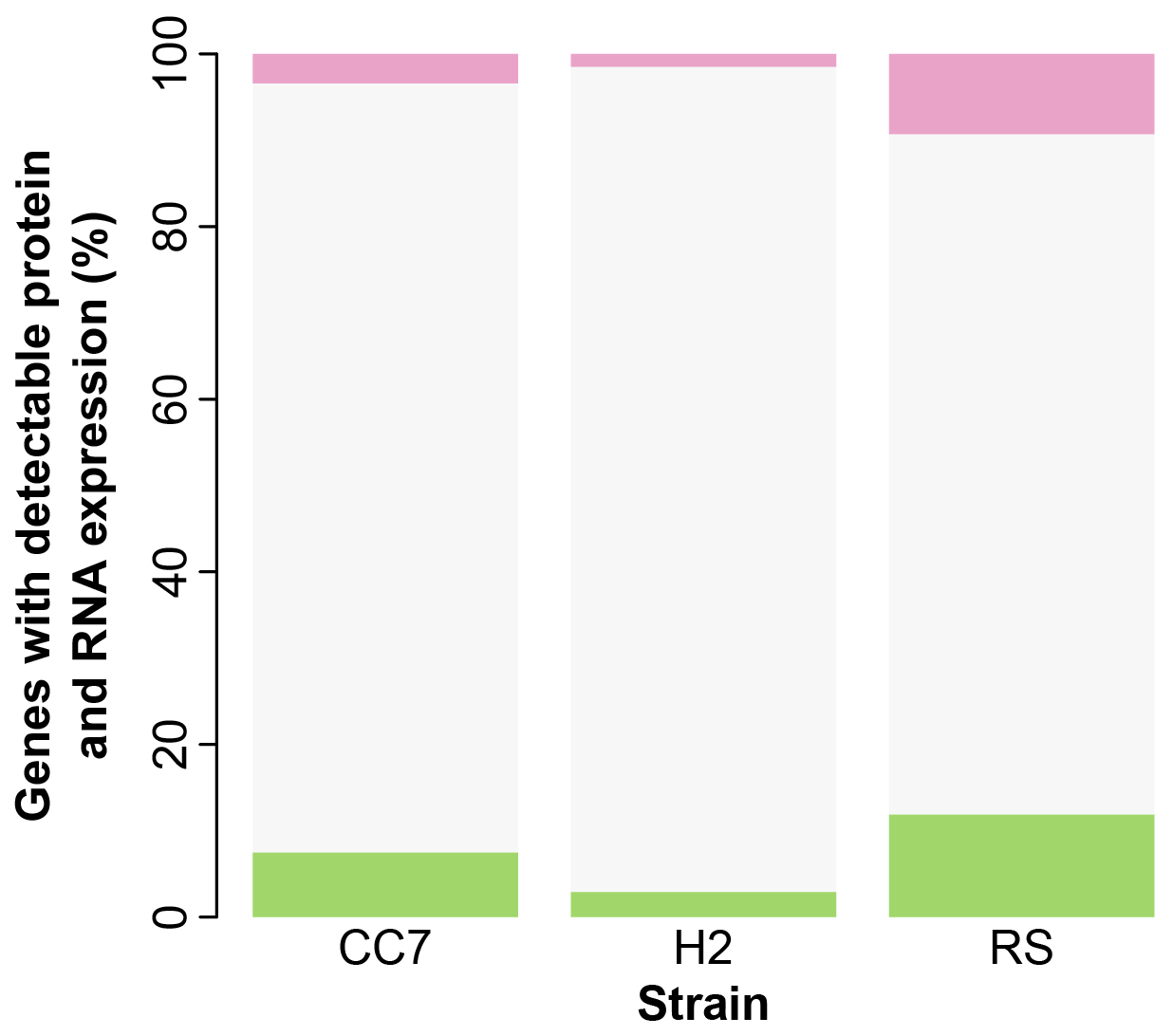
**

**Figure S3. Percentage of significantly differentially abundant proteins matched with expression of respective transcript.** mRNA fold changes were not necessarily significant. Matches were categorized into three groups: differential expression of proteins and transcripts are in concordant directions (green), discordant directions (pink), and those with transcripts that are not differentially expressed (grey)

**Table S1: Clustering of ITS2 sequences from Red Sea Aiptasia (*n* = 4) at 97% similarity.** A total of 348,488 MiSeq sequences (length ≥ 200 bp) were clustered using cdhit-est into 73 clusters. The longest sequence in each cluster, labelled as the cluster representative by cdhit-est, were annotated via BLASTN against NCBI nt. Shown here are subclades of *Symbiodinium* that best matched the top 10 clusters with the highest number of reads. Sources of strains are provided whenever possible; otherwise, links to the sequences (on NCBI) are provided.

| **RS (from Red Sea)** | | | | |
| --- | --- | --- | --- | --- |
|  |  |  | **BLASTN results** | |
| **Sequence ID of cluster representative** | **# reads** | **Percentage** | **Best matching strain** | **Subclade** |
| M02112:179:000000000-B59R4:1:2101:6124:3416 | 109988 | 31.56% | CCMP2456 [1] | A4 |
| M02112:179:000000000-B59R4:1:1116:22509:5885 | 80224 | 23.02% | CCMP2467 [2] | A1 |
| M02112:179:000000000-B59R4:1:1109:22536:2980 | 53807 | 15.44% | voucher 704 [3] | B1 |
| M02112:179:000000000-B59R4:1:2106:18652:11185 | 50243 | 14.42% | RT-89 [4] | A2 |
| M02112:179:000000000-B59R4:1:1103:25367:6720 | 48892 | 14.03% | "clade A4" [5] | A4 |
| M02112:179:000000000-B59R4:1:1101:6462:18873 | 1050 | 0.30% | CCMP2467 | A1 |
| M02112:179:000000000-B59R4:1:1102:3136:16588 | 791 | 0.23% | JCUSG-1 [6] | A2 |
| M02112:179:000000000-B59R4:1:1109:17670:10363 | 537 | 0.15% | multiple best hits [7] | A4/A13 |
| M02112:179:000000000-B59R4:1:1103:9884:3854 | 341 | 0.10% | RT-89 | A2 |
| M02112:179:000000000-B59R4:1:1101:12494:24248 | 297 | 0.09% | CCMP2467 | A1 |
|  |  |  |  |  |
| **CC7 (from Florida Keys)** | | | | |
| **Clade** | **Subclade** | **Citation** |  |  |
| A | A4 | [8] |  |  |
| A, B | A4, B1 | [9] |  |  |
|  |  |  |  |  |
| **H2 (from Hawaii)** | | | | |
| **Clade** | **Subclade** | **Citation** |  |  |
| B | B1 | [10] |  |  |
| B | B1 | [9] |  |  |

[1]: <https://ncma.bigelow.org/ccmp2456>
[2]: <https://ncma.bigelow.org/ccmp2467>
[3]: <https://www.ncbi.nlm.nih.gov/nuccore/344227688/>
[4]: <https://www.ncbi.nlm.nih.gov/nuccore/344227729/>
[5]: <https://www.ncbi.nlm.nih.gov/nuccore/325514313/>
[6]: Onodera et al., 2004 Zooxanthellactone, a Novel γ-Lactone-type Oxylipine from Dinoflagellates of Symbiodinium sp.: Structure, Distribution, and Biological Activity. *Biosci. Biotechnol. Biochem.* **68**(4), 848–852.
[7]: <https://www.ncbi.nlm.nih.gov/nuccore/344227725/>, <https://www.ncbi.nlm.nih.gov/nuccore/325514313/>
[8]: Bieri et al., 2016 Relative Contributions of Various Cellular Mechanisms to Loss of Algae during Cnidarian Bleaching. *PLoS One* **11**(4), e0152693.
[9]: Thornhill et al., 2013 Population genetic data of a model symbiotic cnidarian system reveal remarkable symbiotic specificity and vectored introductions across ocean basins. *Mol Ecol.* **22**(17):4499-515.
[10]: Xiang T., Hambleton E.A., DeNofrio J.C., Pringle J.R., Grossman A. 2013 Isolation of clonal axenic strains of the symbiotic dinoflagellate Symbiodinium and their growth and host specificity. J Phycol **49**, 447-458.

**Table S2: Summary of differentially expressed genes relating to oxidative stress response in CC7, H2 and RS.** All genes with the same annotation were considered. Number of significantly differently expressed homologs in each anemone strain were recorded and summed. Genes are specific towards heat stress and antioxidant response, highlighting stronger transcriptional response in CC7 and RS.

| **Gene annotation** | **Number of homologs** | **Number of genes differentially expressed** | | |
| --- | --- | --- | --- | --- |
|  |  | **CC7** | **H2** | **RS** |
| Cytochrome p450 | 33 | 2 | 3 | 8 |
| Ferritin | 2 | 0 | 0 | 1 |
| Glutathione peroxidase | 7 | 3 | 2 | 3 |
| Glutathione S-transferase | 21 | 2 | 0 | 6 |
| HSP 70 | 6 | 1 | 0 | 3 |
| HSP 90 | 1 | 1 | 1 | 1 |
| NADH dehydrogenase | 30 | 11 | 0 | 2 |
| Nuclear factor erythoid-2 (Nrf2) | 1 | 0 | 0 | 1 |
| Peroxidasin | 19 | 0 | 2 | 5 |
| Superoxide dismutase | 7 | 2 | 0 | 4 |
| Thioredoxin reductase | 4 | 1 | 0 | 3 |
| Total | 131 | 23 | 8 | 37 |

**Table S3: Transcriptome enriched GO-terms shared between CC7, H2 and RS in response to heat stress.** GO-terms were separated into their three categories. Shared terms indicate a core heat stress response common among anemones strains in response to elevated temperatures.

| **GO Category** | **GO Term** | **Annotation** |
| --- | --- | --- |
| **Biological process** | GO:0006457 | protein folding |
|  | GO:0055114 | oxidation-reduction process |
|  | GO:0044267 | cellular protein metabolic process |
|  | GO:0061077 | chaperone-mediated protein folding |
|  | GO:0000381 | regulation of alternative mRNA splicing, via spliceosome |
|  | GO:0000413 | protein peptidyl-prolyl isomerization |
|  | GO:0048025 | negative regulation of mRNA splicing, via spliceosome |
|  | GO:0030199 | collagen fibril organization |
| **Cellular component** | GO:0005788 | endoplasmic reticulum lumen |
|  | GO:0042470 | Melanosome |
| **Molecular function** | GO:0051082 | unfolded protein binding |
|  | GO:0004656 | procollagen-proline 4-dioxygenase activity |
|  | GO:0004222 | metalloendopeptidase activity |
|  | GO:0000166 | nucleotide binding |
|  | GO:0005506 | iron ion binding |
|  | GO:0042605 | peptide antigen binding |
|  | GO:0031418 | L-ascorbic acid binding |
|  | GO:0003755 | peptidyl-prolyl cis-trans isomerase activity |
|  | GO:0009055 | electron carrier activity |
|  | GO:0003756 | protein disulfide isomerase activity |
|  | GO:0005332 | gamma-aminobutyric acid:sodium symporter activity |

**Table S4: Normalized symbiont density (*n* = 4 per-strain-temperature combination).** Units are counts [ml filtrate]^-1^ [g *Aiptasia* protein]^-1^. No significant changes in symbiont counts were detected in any of the three strains (two-tailed *t*-test).

| **Strain** | **25 °C** | | | | **32 °C** | | | | **Two-tailed *t*-test *p*** |
| --- | --- | --- | --- | --- | --- | --- | --- | --- | --- |
|  | **rep 1** | **rep 2** | **rep 3** | **rep 4** | **rep 1** | **rep 2** | **rep 3** | **rep 4** |  |
| CC7 | 13435.92 | 10703.86 | 10314.42 | 10994.14 | 17107.03 | 20738.68 | 10152.22 | 28091.69 | 0.091 |
| H2 | 17327.07 | 11896.7 | 9987.8 | 9090.555 | 14775.69 | 14567.55 | 11997.39 | 13656.53 | 0.424 |
| RS | 21073.79 | 12416.41 | 17881.39 | 36986.34 | 18816.05 | 12128.41 | 16573.64 | 13139.73 | 0.255 |
